# Prevalence of *Ehrlichia-, Babesia*-, and *Hepatozoon*-infected brown dog ticks in Khon Kaen Province, Northeast Thailand

**DOI:** 10.14202/vetworld.2022.1699-1705

**Published:** 2022-07-20

**Authors:** Chatanun Eamudomkarn, Opal Pitaksakulrat, Parichart Boueroy, Sirikanda Thanasuwan, Nattaya Watwiengkam, Atchara Artchayasawat, Thidarut Boonmars

**Affiliations:** 1 Department of Parasitology, Faculty of Medicine, Khon Kaen University, Khon Kaen 40002, Thailand; 2 Cholangiocarcinoma Research Institute, Khon Kaen University, Khon Kaen 40002, Thailand; 3 Department of Community Health Faculty of Public Health, Kasetsart University Chalermphrakiat Sakon Nakhon Province Campus, Sakon Nakhon 47000, Thailand; 4 Department of Veterinary Technology, Faculty of Agriculture Technology, Kalasin University, Kalasin 46000, Thailand; 5 Veterinary Clinic Research Unit, Faculty of Veterinary Sciences, Mahasarakham University, Maha Sarakham 44000, Thailand

**Keywords:** brown dog tick, *Rhipicephalus sanguineus*, tick-borne diseases, tick-borne pathogens

## Abstract

**Background and Aim:**

The brown dog tick, *Rhipicephalus sanguineus* sensu lato, is the most common tick found on domestic dogs in Southeast Asia, including Thailand. Canine tick-borne pathogens are a public health concern worldwide. Tick-borne diseases are diagnosed by identifying pathogens based on the morphological or molecular analyses of dog blood samples. However, the collection of ticks, a non-invasive procedure, is easier than drawing blood. This study aimed to demonstrate the usefulness of collecting brown dog ticks for the diagnosis of tick-borne diseases and for estimating the prevalence of tick-borne pathogens among companion dogs in Khon Kaen, Northeast Thailand.

**Materials and Methods:**

Seventy brown dog ticks from 70 companion dogs in Khon Kaen Province, Thailand, were evaluated for molecular evidence of tick-borne pathogens, including *Babesia* spp., *Ehrlichia canis*, and *Hepatozoon canis*. Ticks were collected from dogs at a private animal hospital based on the presence of at least one of the three inclusion criteria: fever, anorexia, or lethargy. Molecular diagnosis was performed using conventional polymerase chain reaction for the detection of pathogens.

**Results:**

Of the 70 ticks collected from 70 sick dogs, 55 (78.57%) were positive for tick-borne pathogens. The most common infection was a single infection with *H*. *canis* (65.71%) followed by *Babesia* spp. (31.43%) and *E. canis* (30.00%). Coinfection was observed in 14 ticks (20.00%), and coinfection with *Babesia* spp. and *E. canis* was the most prevalent double infection (n = 6). The prevalence of coinfection was identical for *H. canis* mixed with *Babesia* spp. and *H. canis* mixed with *E. canis* (n = 4).

**Conclusion:**

The present study showed that tick-borne pathogens are highly prevalent among companion dogs in Khon Kaen Province. Therefore, we encourage an increase in tick control or the reduction and prevention of tick-borne diseases in this region. Furthermore, this study revealed that ticks are valuable samples for the molecular detection of tick-borne pathogens.

## Introduction

Ticks are blood-feeding vectors that transfer various pathogens to humans and animals and are the most important vectors of animal diseases worldwide [1]. Tick-borne pathogens include a wide range of viruses, bacteria, rickettsia, and protozoa, accounting for more than 100,000 cases of infection in humans worldwide; moreover, they are important causes of diseases in domestic and wild animals [2]. *Rhipicephalus sanguineus* sensu lato is the most common tick found on domestic dogs in Southeast Asia (Cambodia, Lao PDR, Peninsular Malaysia, Myanmar, Thailand, and Vietnam) [3]. *R. sanguineus* populations can reach high densities in regions where dogs are commonly found. The pathogens responsible for canine tick-borne diseases (e.g., *Anaplasma phagocytophilum*, *Ehrlichia canis*, and *Rickettsia* spp.) are of major zoonotic concern and represent an emerging worldwide public health concern for pets and their owners. Among the tick-borne pathogens, *Babesia* spp., *E. canis*, *Hepatozoon* spp., *A. platys*, and *Mycoplasma* spp. are common vector-borne pathogens that are detected in dogs in Southeast Asia and are a major cause of morbidity and mortality [4–8].

The diagnosis of these tick-borne pathogens is an issue for veterinarians because similar clinical signs are observed following infection of various pathogens. Moreover, mixed infections may lead to overlapping or atypical clinical signs [9]. Canine tick-borne diseases can cause subclinical or clinical symptoms, usually associated with anorexia, fever, weight loss, icterus, and anemia, resulting in lethal outcomes [3, 4, 6]. The standard diagnoses of tick-borne diseases are established on the basis of morphological identification of pathogens in blood smears under microscopic examination. Nevertheless, this technique often shows limited sensitivity, is time-consuming, requires expertise, and cannot rely on similar morphology, particularly in mixed infections [9, 10]. Serology-based diagnosis has been widely used; however, the cross-reactions of the techniques and active infection status are drawbacks of this approach. Therefore, molecular techniques are more reliable and useful in diagnosing infections, particularly in cases of subclinical or light infections.

Several studies have been conducted on tick-borne diseases in Thailand [6, 8, 11, 12]. However, these diseases were diagnosed based on the morphological identification of pathogens or molecular diagnosis using blood samples collected from dogs. In Thailand, the detection of the DNA of pathogens in animal ticks has been previously used in epidemiological surveys and taxonomic studies [13–15]. Moreover, the previous report on tick-borne pathogens in dogs in Khon Kaen Province from our study was based only on blood samples for the detection of *E. canis*, *Babesia* spp., and *Hepatozoon canis* [11].

Therefore, this study aimed to define the usefulness of brown dog ticks (*R. sanguineus*) for the diagnosis of tick-borne diseases caused by *Babesia* spp., *E. canis*, and *H. canis*. This is because the collection of ticks is easier than drawing blood and is a non-invasive procedure. Thus, we report the prevalence of tick-borne pathogens in companion dogs in Khon Kaen, Northeast Thailand.

## Materials and Methods

### Ethical approval

The study protocol was approved by the Institutional Animal Care and Use Committee of Khon Kaen University, based on the Ethics of Animal Experimentation of the National Research Council of Thailand (Reference No. 0514.1.75/99).

### Study period, area, and sample collection

**Figure-1:**
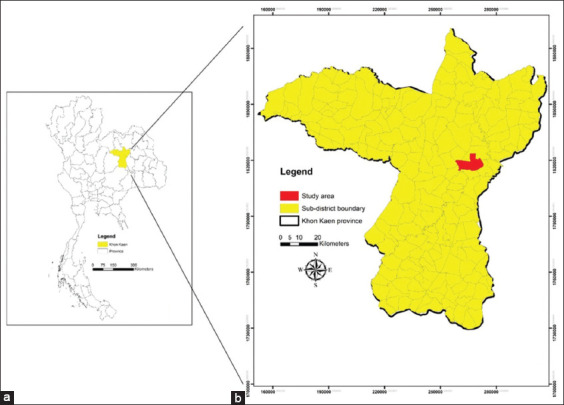
(a) Map of Thailand and the region of Khon Kaen Province with (b) the indication of subdistrict of the study area [Source: The administrative boundaries of Khon Kaen Province and Thailand were downloaded from the DIVA-GIS database. A geographical information system software packages ArcGIS 10.XX was used to create a study map].

Ticks were collected from any area of the bodies of unhealthy dogs at a private animal hospital from January to June, 2018 in Khon Kaen Province, Northeast Thailand (Figure-1). All study dogs were Thai local and mixed breed and had at least one of the three inclusion criteria: fever, anorexia, or lethargy. The age of the study dogs was 1–5 years. The body condition score (BCS) of the dogs was evaluated by palpating and observing fat deposits under the skin according to a 9-point system [16]. The BCS of the dogs in this study was 2–3 points. One tick per dog was collected by handpicking. All ticks on the dog’s body fed on the blood of the same dog. Therefore, if a dog was infected with tick-borne pathogens, all ticks were prone to be infected with the same pathogens. Hence, one tick is representative of all ticks collected from a dog. Moreover, a minimum of one tick is required to check whether only one tick from unhealthy dogs can be useful for diagnosis. A total of 70 ticks from 70 dogs were identified by morphological methods according to taxonomic keys [5, 17, 18] and stored at −20^o^C until they were used for DNA extraction.

### DNA extraction from ticks

DNA was extracted from frozen tick samples using the phenol-chloroform method. Briefly, ticks were washed with sterilized distilled water to remove microorganisms on the surface of the ticks. Next, phosphate-buffered saline (pH = 7.4, Vivantis, Malaysia) was added to the eppendorf tube. The ticks were dissected and crushed using a micropestle. A total of 400 μL of TE buffer master mix (Vivantis) (final concentration of 0.2 mg/mL proteinase K solution (Qiagen, Germany), 1% sodium dodecyl sulfate (Vivantis), and TE buffer in a final volume of 400 μL of master mix solution) were added, followed by incubation for 2 h in a water bath at 56°C. DNA was extracted by adding 400 μL of phenol: chloroform (1:1) mixture, which was centrifuged at 16,099× *g* for 10 min at 8°C. The supernatant was then transferred to a new tube containing 3M sodium acetate (Emsure, Germany) (1/10 v/v) and absolute cold ethanol (RCI Labscan™, Thailand) (2.5 V). This solution was stored at −20^o^C for 30 min and then centrifuged at 16,099× *g* for 10 min at 4°C. The supernatant was discarded. The DNA pellet was washed with 75% ethanol and centrifuged at 16,099× *g* for 10 min at 4°C. The pellet was then air-dried and dissolved in 20 μL of deionized water (deionized water was produced from deionized water system machine at the Faculty of Medicine, Khon Kaen University). Two microliters of the extracted DNA were used for conventional polymerase chain reaction (PCR).

### PCR assay

*Babesia* spp., *E. canis*, and *H. canis* were detected using conventional PCR using specific primers. Primers for *Babesia* spp. and *H. canis* were designed according to the published 18S ribosomal RNA gene sequences as follows: Bab, 5′–CAGGGCTAATGTCTTGTAATTGG–3′ and 5′–ATTTCTCTCAAGCTCCTGAAGG–3′ (GenBank accession no. JQ613105); and Hepcanis, 5’–TTAACGGGGGATTAGGGTTC–3’ and 5’–CGGCCTGCTAGAAACA CTCT–3′ (GenBank accession no. AF176835.1). The PCR amplicon sizes were 557 and 437 bp for *Babesia* spp. and *H. canis*, respectively. The primers for *E. canis* were designed based on *virB9* gene sequences (GenBank accession no.: AY205342.1) as follows: *E. canis*, 5’–CCATAAGCATAGC TGATAACCCTGTTACAA–3’ and 5’–TGGATAATAAAACCGTACTATGTATGCTAG–3’; resulting in an amplicon with a size of 380 bp [11].

The PCR assays were performed in a final volume of 20 μL consisting of 2 μL of 5 μM of each primer, 2 μL of 25 mM MgCl_2_, 2 μL of 5 mM dNTP, 2 μL of extracted DNA, 2 μL of 10× buffer, 0.08 μL of 5 U/μL of DNA Taq polymerase (RBC Bioscience, Taipei, Taiwan), and nuclease-free water added up to the final volume. Reactions were performed using a GeneAmp PCR System C1000 thermal cycler (Bio-Rad, Hercules, CA). The thermocycling conditions included an initial denaturation at 94°C for 5 min; followed by 35 cycles of 94°C for 30 s, annealing at 63°C for 1 min, and extension at 72°C for 1 min; and a final extension at 72°C for 5 min. The PCR products were analyzed using electrophoresis in a 2% agarose gel (Vivantis) stained with 0.1 mg/mL ethidium bromide (Bio-Rad, USA), and the gel was visualized using a gel documentation system (Bio-Rad). Each set of experiments included negative and positive controls. Nuclease-free water (Water was produced from water system machine at the Faculty of Medicine, Khon Kaen University) replaced DNA templates in the negative controls. A specific band for each species was excised from the agarose gel and sequenced for species confirmation.

### Statistical analysis

Data were recorded in Microsoft Excel and imported into IBM SPSS Statistic Version 23.0 (IBM Corp., NY, USA) for statistical analysis. Descriptive statistics and the Chi-square test were used to analyze the data. The results were considered statistically significant at p < 0.05. A Venn diagram was created using Microsoft Excel.

## Results

### Prevalence of tick-borne pathogens in ticks

**Figure-2:**
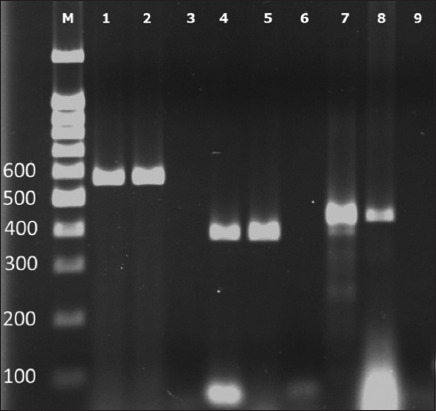
The conventional polymerase chain reaction result for specific primer analysis. Each set of experiments included negative and positive controls. Lanes 1–3 were an experiment assay for *Babesia* spp. Lanes 4–6 were assayed for *Ehrlichia canis*, and lanes 7–9 were for *Hepatozoon canis*. The negative controls of each experiment are shown in lanes 3, 6, and 9. The positive controls of each experiment are shown in lanes 2, 5, and 8. Positive DNA samples for *Babesia* spp., *E. canis*, and *H. canis* are shown in lanes 1, 4, and 7, respectively (M; marker, DNA size markers were shown in number 100–600 refer to molecular weight 100–600 bp).

**Figure-3:**
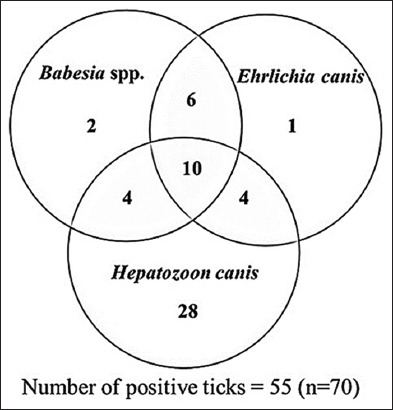
The distribution of tick-borne diseases in brown dog ticks (n = 70).

One tick was collected from each dog, resulting in the collection of 70 ticks from 70 dogs. The DNA of tick-borne pathogens from tick samples was detected using conventional PCR according to each primer. The amplicon sizes of *Babesia* spp., *E. canis*, and *H. canis* were 557, 380, and 437 bp, respectively, as shown in Figure-2. The DNA of tick-borne pathogens was detected in 55 of the 70 ticks (78.57%), as presented in Table-1. The most prevalent tick-borne pathogen was *H*. *canis* (n = 46) followed by *Babesia* spp. (n = 22) and *E. canis* (n = 21). Triple infection with *H*. *canis*, *Babesia* spp., and *E. canis* was observed in 10 dogs (14.3%). Double infection with each pathogen was also detected, as shown in Table-1. Mixed infection with *Babesia* spp. and *E. canis* was the most prevalent double infection (n = 6), as indicated in the Venn diagram (Figure-3). The prevalence of mixed infection was identical between *H. canis* mixed with *Babesia* spp. and *H. canis* mixed with *E. canis* (n = 4), as shown in Figure-3. Among the 70 ticks examined, 15 (21.43%) were negative for all pathogens. The positive samples of each species were randomly selected and processed for DNA sequencing to confirm the tick-borne pathogen species, which showed 100% identity with *Babesia* spp., *H*. *canis*, and *E. canis*.

**Table 1:** The prevalence of tick-borne pathogens in 70 dog ticks in Khon Kaen Province.

Pathogens	Positive (n)
*Babesia* spp.	22 (31.43%)
*Ehrlichia canis*	21 (30.00%)
*Hepatozoon canis*	46 (65.71%)
*Babesia* spp. + *Ehrlichia canis*	6 (8.57%)
*Babesia* spp. + *Hepatozoon canis*	4 (5.71%)
*Ehrlichia canis +* H*epatozoon canis*	4 (5.71%)
*Babesia* spp. + *Ehrlichia canis +* H*epatozoon canis*	10 (14.3%)
Negative for all three pathogens	15 (21.43%)

## Discussion

This study showed that tick-borne pathogens could be detected in ticks collected from unhealthy dogs at an animal hospital. All study dogs were defined as unhealthy with at least one of the three inclusion criteria: fever, anorexia, or lethargy (common symptoms associated with tick-borne diseases). The dogs included in this study were Thai local and mixed breed with a BCS of 2–3. The previous studies have shown that the breed is not associated with tick infection and the presence of canine tick-borne diseases [19–21]. A survey study conducted in Panama for several pathogens and zoonotic parasites in dogs indicated no correlation between BCS and infection with zoonotic parasites [22]. Our study revealed that tick-borne pathogens were highly prevalent among companion dogs in Khon Kaen Province. In 2016, the survey data of the Bureau of Disease Control and Veterinary Unit, Department of Livestock Development, Thailand, showed that the dog population in Thailand is approximately 7.3 million; of these, 750,000 are stray dogs. Due to a large population of stray dogs throughout Thailand, a much higher rate of tick-borne pathogens is expected because of their greater exposure to ticks.

The prevalence obtained in this study differed from that of a previous report based on blood samples collected from dogs, which found a higher prevalence of *Babesia* spp. in the Khon Kaen region [11]. However, the previous studies conducted in Thailand reported surveys of canine tick-borne diseases with different infection rates in each region; Piratae *et al*. [12] found that *E. canis* showed the highest infection rate in Mahasarakham Province, which is a neighboring province to Khon Kaen Province followed by *H. canis* and *B. canis*. A study conducted in the central part of Thailand using blood samples collected from 20 sick dogs found that all dogs were negative for *Babesia* infections [23]. PCR on blood samples collected from one stray dog for the detection of canine vector-borne diseases in the southern part of Thailand revealed that *Mycoplasma* spp. was the most common single infection, followed by *Hepatozoon* spp. [6]. Recently, Juasook *et al*. [24] reported that *E. canis* was the most common canine tick-borne pathogen in Thailand and was detected in either sick or healthy dogs and brown dog ticks. Regarding mixed infections, our study results agree with those of the previous studies that suggested that dogs in Thailand have extensive exposure to vector-borne diseases and that coinfection with these pathogens is common [6, 24, 25]. Similar studies were also conducted in other Southeast Asian countries as per the followings; A recent serological survey of canine vector-borne diseases in an animal shelter in Malaysia found that *Ehrlichia* spp. was the most prevalent pathogen (44.7%), followed by *Anaplasma* spp. (30.1%) and *Dirofilaria immitis* (13.6%) [26]. A study conducted in the Philippines using PCR of blood samples found that hepatozoonosis and babesiosis were the most prevalent infections (5.3%) followed by ehrlichiosis (4.4%) and anaplasmosis (3.5%) [27]. The most prevalent canine vector-borne pathogen found in Cambodia is *B. vogeli* (32.7%), followed by *E. canis* (21.8%), *D. immitis* (15.8%), *H. canis* (10.9%), and *Mycoplasma haemocanis* (9.9%) [4]. Data collected from European countries revealed that the prevalence of *E. canis* ranged from 0.3% to 50.0% in different countries [28]. A retrospective study of ehrlichiosis in dogs from North Carolina and Virginia reported a mortality rate of 18.0% [29]. The mortality rate of canine babesiosis in an academic hospital setting in South Africa was approximately 5.0% [30]. Moreover, a high prevalence of *H. canis* was detected among domestic dogs (63.3%) in Mexico [31]. These variations in prevalence and inconsistencies among different countries can be attributed to various factors, such as different locations, ecological variations, travel history of animals, and the study population [32, 33].

The gold standard approach for blood parasite diagnosis is the microscopic examination of blood smears. However, this technique is a time-consuming process that requires expertise and has low sensitivity; furthermore, due to similar clinical characteristics of other pathogens, this technique leads to misdiagnoses of infections, particularly in mixed infections [9, 11, 24, 25]. Alternatively, diagnostic test kits based on immunological techniques have been widely used in this setting; however, the presence of cross-reactions and previous infection is a challenge in this technique [12, 34]. Therefore, a molecular diagnosis based on blood samples has been examined and proven to confirm tick-borne diseases in numerous studies. Furthermore, the direct examination of blood smear is not sufficient in the case of coinfection with two or more pathogens [9, 35]. Nevertheless, molecular techniques require equipment and have a relatively high cost compared with microscopic examination of blood smears. Thus, there is a need to develop techniques for diagnosis during the acute stage of infection when treatments are most effective, as delays in diagnosis can increase the cost of treatment. A study on human Lyme disease conducted in the United States found that the cost of treating late-stage Lyme disease is 12 times higher than the cost of early treatment, which is around $24,000/year [36]. Moreover, the calculation of the financial impact of canine vector-borne diseases on the veterinary health-care division yielded an amount of $80.5–$177 million USD annually, and the cost of treatment may show a gradual change every year [37]. Therefore, an early and accurate diagnosis of these infections can lead to appropriate and early treatments, which may promote cost-related savings for pet owners. Thus, an appropriate diagnostic technique is required. Blood drawing is an invasive procedure, whereas the collection of ticks is an easier, non-invasive procedure. Therefore, the detection of the DNA of tick-borne pathogens in ticks could be useful for diagnosing tick-borne diseases.

The prevalence of the main host of *R. sanguineus* in domestic dogs and the mean intensity of tick infestation varied according to several factors, for example, dog population density and the proportion of dogs treated with ectoparasiticides within a population [38]. Moreover, climate change is one of the greatest threats to humans and animals in the 21^st^ century, affecting vector biology and disease transmission. Several studies have discussed climate change and its impact on tick-borne diseases worldwide [38–42]. Warming of the global temperature directly impacts vector-borne diseases, which affect vector development, vector physiology, and vector–host–pathogen interactions [40, 42], thereby increasing the tick populations and tick-borne pathogens.

Furthermore, recent studies have reported that a tick exposed to a higher temperature may bite an unusual host [43]. This result suggests that humans living in regions with warmer temperatures and/or longer summers will be at a higher risk of human parasitism by *R. sanguineus*, thus increasing the risk of transmission of zoonotic diseases [38]. Therefore, tick-borne infection is likely to increase under climate change conditions.

However, in the present study, the criteria used to define sick dogs decisively created a selection bias toward diagnosing tick-borne diseases. The prevalence of these diseases in the animal hospital population was high, which suggests a widespread exposure to tick-borne pathogens among dogs in Thailand. Therefore, we encourage an increase in tick control or the reduction and prevention of tick-borne diseases in this region. Moreover, dogs have often been considered effective indicators for assessing the risk of human infection [44, 45].

Our results indicate that tick samples are useful for the molecular detection of tick-borne pathogens. However, a limitation of this study was that the results were based only on the detection of pathogens in dog ticks. Furthermore, this study did not address the severity of the clinical signs and symptoms and the duration of the infection associated with the pathogen. Therefore, future studies should analyze and compare the detection of tick-borne pathogens in tick and blood samples using molecular techniques and morphological identification to achieve greater sensitivity and specificity. Once molecular detection techniques have been established in tick samples, it can be useful in small animal hospitals and for the prevention and control of tick-borne diseases in field studies. Moreover, these techniques can be applied to vector-borne diseases, such as those associated with flea. Finally, these findings can lead to the study of other tick species and tick-borne diseases in other household animals, environments, and human populations in Thailand, thereby improving our understanding of the animal hosts, tick species, and clinical symptoms associated with pathogens.

## Conclusion

To the best of our knowledge, the present study is the first report on the detection of the DNA of tick-borne pathogens in ticks collected from dogs in Khon Kaen Province, Northeast Thailand. Our results revealed that ticks could be a valuable sample for the molecular diagnosis of tick-borne pathogens. In this study, *H. canis* was the most prevalent pathogen, followed by *Babesia* spp. and *E. canis*, and a high prevalence of coinfection was detected. This suggests a widespread exposure to tick-borne pathogens among dogs in Thailand. Further studies using additional tick samples and analyses of the duration of infection and degree of severity of these diseases are needed to determine the applicability of the diagnosis based on the presence of pathogenic DNA in dog ticks. This technique can be useful in small animal hospitals and can be assessed as a surveillance tool for tick-borne diseases. The awareness of tick-borne pathogens in animals and humans and their distribution will lead to the development of better plans to prevent and control zoonotic tick-borne diseases. However, microscopic examination alone, which results in an underestimation of their prevalence, is not appropriate for diagnosing these pathogenic diseases. Therefore, a useful diagnostic technique is of great importance.

## Authors’ Contributions

CE and TB: Designed the study. CE, PB, and AA: Performed the experiments. CE, OP, ST, and NW: Coordinated sample collection. CE: Analyzed the results and drafted the manuscript. TB: Supervised the study. All authors have read and approved the final manuscript.
